# A visual step-by-step guide for clinicians to use video consultations in mental health services: NHS examples of real-time practice in times of normal and pandemic healthcare delivery

**DOI:** 10.1192/bjb.2020.71

**Published:** 2020-12

**Authors:** Gemma Johns, Jacinta Tan, Anna Burhouse, Mike Ogonovsky, Catrin Rees, Alka Ahuja

**Affiliations:** 1 Aneurin Bevan University Health Board, UK; 2 Northumbria Healthcare NHS Foundation Trust, UK; 3 Life Sciences Hub, Welsh Government, UK

**Keywords:** Video consultations, COVID-19, telepsychiatry, mental health, digital health

## Abstract

Despite the increasingly widespread use of video consultations, there are very few documented descriptions of how to set up and implement video consultations in real-time practice. This step-by-step guide will describe the set-up process based on the authors’ experience of two real-time National Health Service (NHS) examples: a single health board use (delivered in normal time), and an All-Wales National Video Consultation Service roll-out (delivered during an emergency pandemic as part of the COVID-19 response). This paper provides a simple visual step-by-step guide for using telepsychiatry via the remote use of video consultations in mental health services, and outlines the mandatory steps to achieving a safe, successful and sustainable use of video consultations in the NHS by ensuring that video consultations fit into existing and new NHS workflow systems and adhere to legal and ethical guidelines.

There is a large and growing evidence base of published data that demonstrates an overall consensus of suitability, acceptability and satisfaction regarding the use of digital technology,^1–6^ particularly video consultations for the purpose of remote assessments and appointments in mental health, known as ‘telepsychiatry’.^7–15^ Telepsychiatry is widely reported to be at least as efficient and effective as traditional face-to-face care, providing improved clinical and quality of life outcomes across a wide range of population groups and settings.^7–20^ Studies have compared video consultations with standard in-person care and concluded that video consultations might be superior to in-person consultations for some forms of treatment and population groups.^2,7^ The evidence base for telepsychiatry remains strong and consistent across mental health studies for both adult and child services.^7–20^ It is commonly argued that mental health and psychiatry are particularly well suited to video consultations and that the psychiatric interaction translates exceptionally well to the technological world. This is because many treatments are based on interpersonal ‘talking therapies’ and medication management, which typically do not require any other medical devices for clinical use, perhaps in contrast to other specialties.^21^ Video consultations have the potential to offer many additional benefits to patients, families and clinicians besides treatment. They are reported to improve and widen patient and family access to healthcare, support co-production and self-management, increase efficiency and improve clinical outcomes, as well as significantly reducing clinical time and patient and family travel.^2,22,23^

## Local evidence to support this step-by-step guide

### The CWTCH quality improvement project

In 2019, the Health Foundation^24^ funded Aneurin Bevan University Health Board (ABUHB) for 1 year to establish a telepsychiatry programme with the objective of providing mental health appointments to children and adolescents within Gwent mental health services. This ABUHB programme is called Connecting with Telehealth to Children in Hospital and Healthcare (CWTCH). It is a National Health Service (NHS) quality improvement project that provides faster and more efficient appointments in child and adolescent mental health services (CAMHS) using a communication platform called Attend Anywhere (https://www.attendanywhere.com). The programme tested the suitability and acceptability of telepsychiatry and measured satisfaction across a wide range of settings and uses, including paediatric wards for emergency assessments, out-patient appointments, medication reviews, autism assessments, school postvention clinics for pupil suicides, virtual groups and more. It demonstrated that telepsychiatry in CAMHS is a highly suitable adjunct to routine ways of working; once people became familiar with this way of holding appointments, it was rated as acceptable and satisfactory by patients, families and clinicians.^25,26^

### Rebranding of CWTCH and Royal College of Psychiatry endorsement

CWTCH has now been rebranded and is called CWTCH Cymru. It has received local and national recognition for its success and is now considered an exemplar of good practice across Wales. CWTCH Cymru and its guiding principles^26^ have also been endorsed by the Welsh Royal College of Psychiatrists.^27^

### Partnership and development of the national roll-out of video consultations

In March 2020, in response to the COVID-19 emergency, CWTCH Cymru went into partnership with Technology Enabled Care (TEC; https://digitalhealth.wales/tec-cymru) and the Welsh Government to form a National Video Consultation Service.^28^ This service is currently rapidly scaling up the routine use of video consultations across Wales, using the Attend Anywhere communication platform, to all appropriate primary, secondary and community care services, including mental healthcare for all ages. The National Video Consultation Service has a fully resourced website, with helpful guides, videos and toolkits, which can be accessed and used in addition to this paper.^28,29^

### Video consultation experience and lessons learned

The experience gained from working on two very different sized projects (small versus large scale) and in two very different contexts (normal versus pandemic) has demonstrated that regardless of the scale and rate of adoption, there are distinct challenges in introducing video consultations as a new way of working in the NHS for clinicians. Clinician acceptance and use of video as an accepted alternative to established ways of working were found to be rate-limiting factors with respect to adoption and spread. This challenge is defined by the authors as ‘clinician need versus clinical need’. This definition suggests that video consultations are more likely to be adopted and accepted as a feasible approach to healthcare delivery when the ‘need’ for this way of working is defined and accepted by the clinician or service, compared with when they are defined and accepted as a clinical need. In other words, video consultations are generally more successful and sustainable when a clinician or service perceives the need themselves and requests the service, rather when they are motivated by the projection of need or want from their patient population. It is therefore essential to establish and define this ‘need’ within a service, seeing clinician/service ‘pull’ for video consultation as an essential criterion for successful adoption. When the perceived need for video consultations by clinicians and services increased in the pandemic context, there was greater willingness to test new ways of working and overcome perceived or internal barriers to change. This has significant implications for how video consultations should be introduced into services and organisations, highlighting the need to focus on both the technical and the cultural aspects of the adoption process. These findings mirror the work of Greenhalgh et al and their ‘NASSS’ (non-adoption, abandonment and barriers to spread, scale-up and sustainability) framework.^30^

## Rationale and objectives

Despite the increasingly widespread use of video consultations, there are very few visual and fully documented descriptions of how to set up and implement such consultations in real-time practice with specific attention to features such as fitting into existing systems and the legal and ethical requirements of video consultations. This step-by-step guide will describe the process based on two examples: a single health board project (delivered in normal time), and an All-Wales National Video Consultation service roll-out (delivered during the COVID-19 pandemic emergency).

## Methods

This paper was designed to be a simple and visual step-by-step guide describing how to set up and use video consultations for mental health services, including a range of ‘technical’ skills and knowledge that clinicians and services may find helpful for the adoption of video consultations. This paper will describe the various steps that have been taken by the authors in their real-time experience to initiate and implement a new video consultation programme in an existing NHS systems. The visuals used on the TEC website and in this paper are adaptations and syntheses of work from other recent video consultation reports in the UK.^31–33^ Note that the visual diagrams and infographics used in this paper are examples based on the communication platform Attend Anywhere (https://www.attendanywhere.com); therefore, descriptions and instructions may differ slightly depending on the type of platform used in your health service.

Ethical approval was obtained from all seven Welsh research and development departments. Consent was obtained from all participants in both CWTCH and the National Video Consulting Service.

## Results

### Step-by-step guide

This section of the paper will provide a step-by-step guide that is divided up into three distinct sections. The first section demonstrates how to set up video consultations in an existing healthcare workflow. The second section provides the appropriate steps regarding ethical and legal principles. The third section discusses how to use video consultations in a secondary care health service, with the help of an infographic diagram.

## A guide to setting up video consultations in existing systems

### Managing the video consultation service

**Fig. 1:**
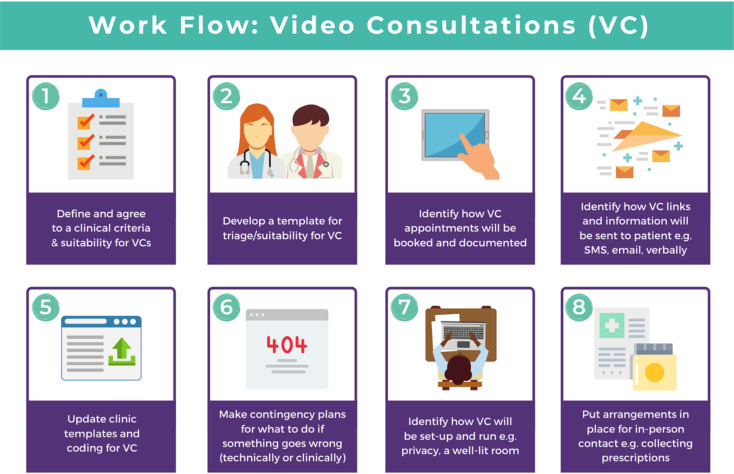
Workflow visual guide. Diagram from the Welsh National Video Consultation Service Toolkit.^29^

Within your healthcare service, you will need to identify a lead clinician who will decide how best to set up, use and manage the video consultations (Fig. 1). This person will be the ‘point of call’ for your service and its use of video consultations.

### Setting clinical criteria

Your healthcare service will need to define and agree on clinical criteria for video consultations and the suitability and appropriateness of appointment types. Following this agreement, a template for triage or suitability would need to be developed and provided to the person(s) in charge of making video appointments, e.g. a receptionist.

### Making and managing appointments:

Your service will need to identify how video consultations will be made, and who will make and deliver these appointments. Your service will also need to identify how appointment links and information will be sent to patients – for example, via a letter, verbally or via an SMS text or email – and how appointment slots will be offered, documented and given to the delivering clinician. In addition, your service will need to decide how the video consultation will be managed, how clinical templates and coding will be used for video consultations, and how these will be matched to existing systems. Your service will need to have a contingency plan for possible scenarios or problems (for both technical and clinical possibilities). Finally, your service will need to consider how clinical information is later documented, for example, similarly to the hand-written notes used in usual practice.

### Setting up the clinical space

Your health service will need to identify how video consultations will be set up. For example, you will need to think about factors such as the room layout, e.g. whether it is well-lit and well-positioned, confidentiality issues and clinical appropriateness. More about this can be found in the following sections.

### Additional considerations

Your service will need to decide how to deal with providing additional information; for instance, if the picture definition obtained via a video consultation is not good enough to allow accurate visualisation and identification of skin lesions, your service needs to decide how to obtain this additional information. It is important that patients are able to receive patient information leaflets as they would in a standard consultation. Your service needs to decide the best mechanism for communicating this information to them. For example, you could email them. Your service would also need to consider additional plans for in-person contact, such as for collection of prescriptions.

## A guide to risk, safeguarding and ethical guidelines

**Fig. 2:**
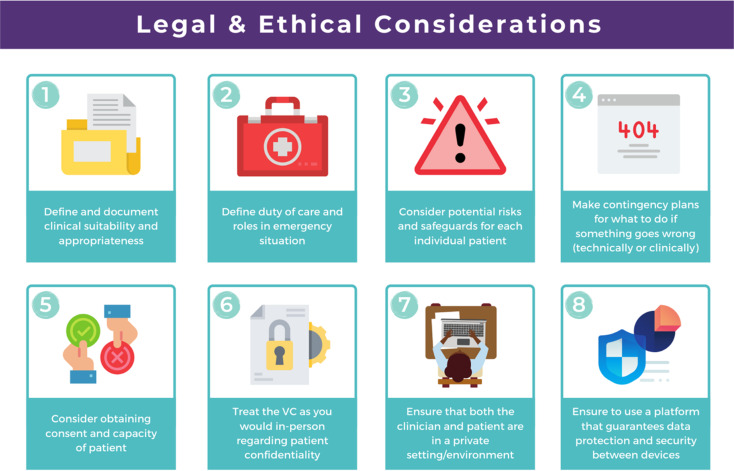
Legal and ethical visual guide. Diagram from the Welsh National Video Consultation Service Toolkit.^29^ VC, video consultation.

Key considerations in the use of video consultations include legal and ethical issues, such as defining and documenting patient suitability and the role of the clinician, risk assessments and contingency planning, privacy, confidentiality, security and consent (Fig. 2). Appropriate strategies covering ethical issues will be necessary, such as taking informed consent and ensuring confidentiality and security while using technology, and procedures for conducting risk assessments.

### Define and document clinical suitability and appropriateness

Your healthcare service will need to define and agree to clinical criteria for video consultations and the suitability and appropriateness of appointment types. This defining and documentation process would need to include the roles and responsibilities of clinical staff.

### Risk, safeguarding and ethical considerations

All types of mental health services using video consultations, regardless of their level of risk, should consider conducting risk assessments, and abide by safeguarding principles and ethical guidelines.

To start thinking about the population your service will deliver a video consultation to, and the risk exposure that may arise, it is advised to conduct an initial ‘process mapping’ of your service. Process mapping will help your team to define video consultations in the context of your service, understand who is responsible for what and where, and to flag up any potential concerns or risk exposures. Process mapping and risk assessment will help your service to start thinking about and understanding what types of risks may arise in specific situations, and possibly how to avoid them.

You would need to think about the following.

• Would using video consultations instead of in-person care increase risk in any way?
• If so, what are these risks, and can they be resolved?
• Is the risk of using video consultations greater than not seeing the patient at all?
• Would these risks be the same if the service was delivered in person?
• What other types of risks might there be – such as the setting, environment and clinical outcome?

On completion of process mapping and initial risk assessments, the next step would be to start thinking about – and formally agreeing to and documenting – clear and concise safeguarding contingency plans for your video consultation service. This would involve a ‘what to do’ plan in the event of an emergency or concern arising during a virtual appointment. It is advised to list a wide range of scenarios, ranging from low- to high-risk possibilities. Make it as specific to your service as possible, to make potential scenarios relatable to your staff. When developing the contingency plan, think about who is best suited to develop it and who will be following it, and consider a wide range of opinions and possibilities. Develop a list of all possible scenarios and all levels of risk exposure, and make them specific, applicable and relatable to your service.

### Ethical guidelines

Video consultations, like any other form of healthcare delivery, will need to be treated exactly the same way as in-person care with regards to ethical guidelines and procedures. However, owing to the obvious remoteness of a virtual appointment, there are additional ethical considerations which need to be considered and applied, such as confidentiality, privacy and security issues.

It is essential that a video consultation service replicates an in-person appointment or assessment as much as possible. For example, the setting of an appointment room would ideally need to be the same as an in-person appointment room, e.g. if your service would normally use a private room for an in-person appointment, then a virtual appointment would also need this. It is also important to ensure that the platform used for video consultations is safe and secure, and that it meets your existing health systems standards, including software encryption. Many popular video chat platforms such as FaceTime and WhatsApp are not compliant with healthcare standards; therefore, you would need to seek out a safe and secure platform such as Attend Anywhere (https://www.attendanywhere.com).

Informed consent is the process of seeking agreement from a person before taking a course of action that requires consent. Informed consent is required from any person who is receiving a video consultation. There are two types of consent.

• Implied consent (or tacit consent), which is signalled by the behaviour of an informed person in agreement. This type of consent is typically used in the delivery of ‘in-person’ healthcare.
• Explicit consent is when a person actively agrees, either verbally or in writing. This type of consent is highly recommended for video consultations, as signalled (implied) behaviour may be more difficult to capture remotely.

To obtain consent, the person giving it would need to be considered to fully understand the process and to have full capacity to do so. A person with incapacity, such as a child or vulnerable adult, may not be able to give informed consent; therefore, parental or guardian consent (known as assent) would be required.

## A guide to conducting the video consultation

**Fig. 3:**
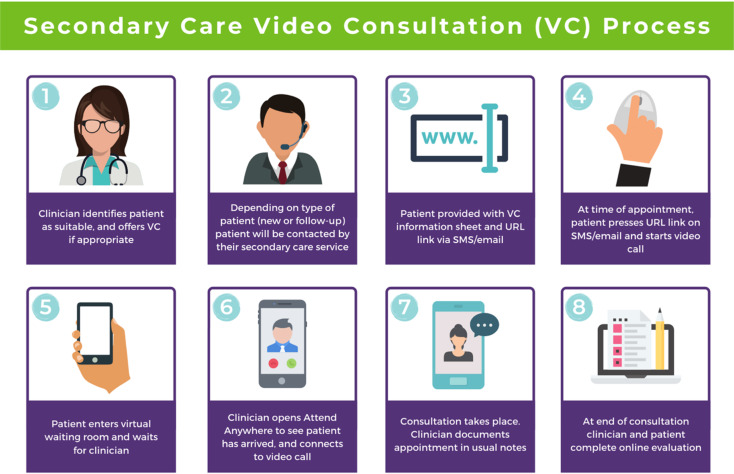
How to use video consultations visual guide. Diagram from the Welsh National Video Consultation Service Toolkit.^29^

The final step of this guide describes how to use video consultations in a secondary healthcare service (Fig. 3).

### Define and document clinical suitability and appropriateness

As shown in the above Figs 1 and 2, your healthcare service will need to define and agree to clinical criteria and patient suitability for video consultations. This is considered the most important step for video consultations.

### Contact the patient and send instructions and video call link

Depending on the type of communication platform (e.g. Attend Anywhere) you will be using, the patient will need to be offered the video appointment; they will then need to agree to it, after which they will need to be sent the patient information sheet and URL link to access the video call.

### Set up video call and start consultation

Again, depending on the type of communication platform you will be using, there will need to be clear steps set out to determine how best to use video consultations in your service.

### Evaluation component

To ensure that the use of video consultations is properly integrated into your health service, it is important to capture feedback on use, acceptability, suitability and satisfaction. It is therefore advisable to establish an evaluation framework to capture this. An example of this may include attaching a basic satisfaction survey to the end of the video consultation, asking a few ‘how did it go’ questions.

**Fig. 4:**
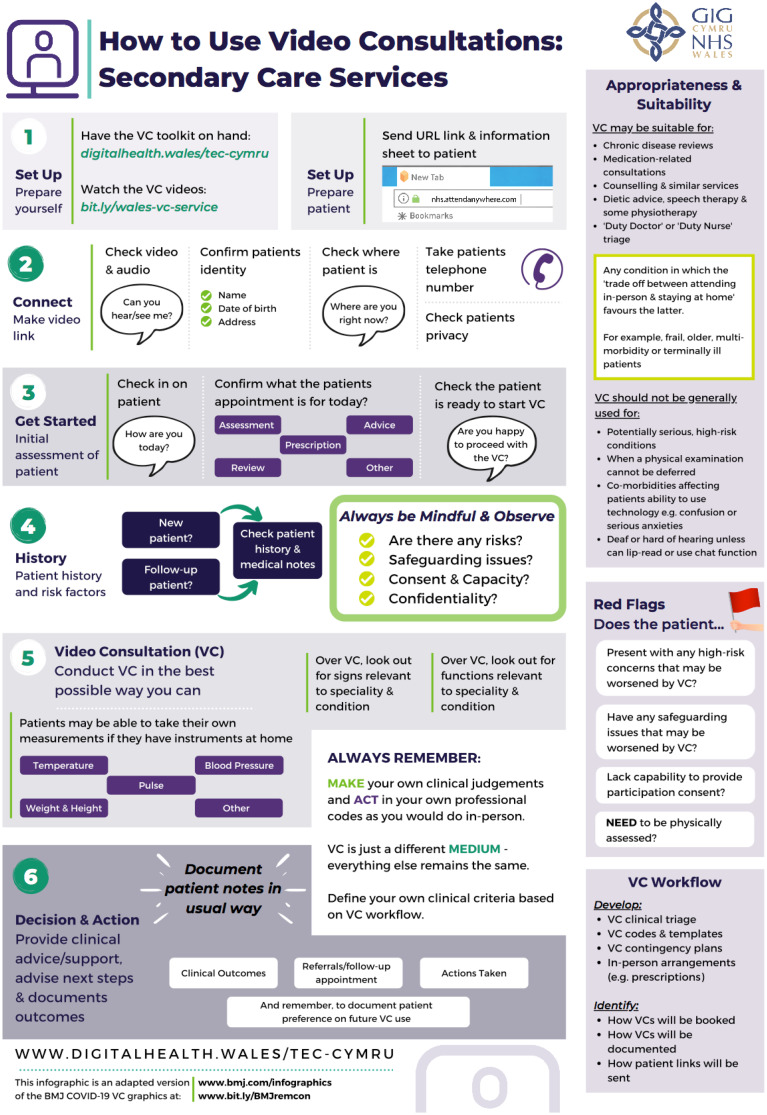
Infographic visual guide: video consultations in secondary care. Diagram from the Welsh National Video Consultation Service Toolkit.^29^

For additional information, please see the TEC website (https://digitalhealth.wales/tec-cymru), which provides a detailed step-by-step guide on setting up a video consultation (Fig. 4), healthcare-specific toolkits, including examples of information sheets, suggested scripting for clinicians, technical guides to the Attend Anywhere communication platform and much more.

## Conclusions

This paper outlines a simple visual step-by-step guide to help clinicians to set up and use video consultations in mental health services. This resource has been used to support clinicians to gain the technical skills and knowledge required to routinely use video consultations in practice. In addition, we found that there is an important ‘cultural’ aspect to successful adoption of video consultations, where the rate-limiting factor for successful adoption is the ‘need’ and ‘pull’ for this way of working to be defined and accepted by the clinician or ser vice.

When the perceived need for video consultations by clinicians and services increased in the pandemic context, there was greater willingness to test new ways of working and overcome perceived or internal barriers to change. It will be interesting to see whether having made the change to this new way working in the pandemic context, clinicians and services actively choose to maintain these newly gained technical skills – and also whether patients and carers, having experienced video consultations for the first time, increase their demand for this to become a new ‘routine’ way of working. As use of video consultations increases, we will also undoubtedly learn how to titrate this offer according to need, circumstance and demographics, and discover which healthcare treatments can best be deployed or augmented through the use of video consultations.

This paper provides a guide to using video consultations in the NHS, based on personal experience of the authors and feedback from their evaluation. However, it is still early days for video consultations in Wales, and more research is needed to understand more about their use, particularly what can and can't be done using video consultation, as this is still unspeculative and unproven.

## About the authors

**Gemma Johns** is Research & Evaluation Lead of CWTCH and TEC Cymru ABUHB, Wales, UK. Gemma worked on both the CWTCH and National Video Consultation project and led the evaluation for both. **Jacinta Tan** is a Child and Adolescent Mental Health Consultant Psychiatrist and Project Manager of CWTCH, ABUHB. **Anna Burhouse** is the Health Foundation QI Adviser for CWTCH and supported the evaluation of the National VC programme, Northumbria Healthcare NHS Foundation Trust. **Mike Ogonovsky** is Assistant Director of Informatics ABUHB and Senior Responsible Officer of TEC Cymru. **Catrin Rees** is at Life Sciences Hub, Welsh Government, and is a Graphics & Web Designer for TEC Cymru. **Alka Ahuja** is a Child and Adolescent Mental Health Consultant Psychiatrist, Clinical Lead for TEC Cymru, Chief Investigator for CWTCH.
