# A visual step-by-step guide for clinicians to use video consultations in mental health services: NHS examples of real-time practice in times of normal and pandemic healthcare delivery – ERRATUM

**DOI:** 10.1192/bjb.2020.144

**Published:** 2022-06

**Authors:** Gemma Johns, Jacinta Tan, Anna Burhouse, Mike Ogonovsky, Catrin Rees, Alka Ahuja

In the original published article, country names were missing from the author affiliations. This has now been corrected.
